# Metagenomic analysis of isolation methods of a targeted microbe, *Campylobacter jejuni*, from chicken feces with high microbial contamination

**DOI:** 10.1186/s40168-019-0680-z

**Published:** 2019-04-25

**Authors:** Junhyung Kim, Jae-Ho Guk, Seung-Hyun Mun, Jae-Uk An, Hyokeun Song, Jinshil Kim, Sangryeol Ryu, Byeonghwa Jeon, Seongbeom Cho

**Affiliations:** 1 Research Institute for Veterinary Science and College of Veterinary Medicine, Seoul National University, Seoul, Republic of Korea; 2 Department of Food and Animal Biotechnology, Department of Agricultural Biotechnology, Research Institute for Agriculture and Life Sciences, and Center for Food and Bioconvergence, Seoul National University, Seoul, Republic of Korea; 3 School of Public Health, University of Alberta, Edmonton, Alberta Canada

**Keywords:** Metagenomics, Microbial community analysis, Isolation method, *Campylobacter jejuni*

## Abstract

**Background:**

Originating from poultry, particularly chickens, *Campylobacter jejuni* is the leading foodborne pathogen worldwide and a major cause of campylobacteriosis. Isolating *C. jejuni* is difficult due to its specific growth requirements, the presence of viable but non-culturable bacteria, and because it is often masked by competing flora. Currently, there is no optimized method for isolating *C. jejuni* from chicken feces. Here, we evaluated the method for isolating *C. jejuni* from chicken feces using culture-independent sequence-based metagenomics and culture-dependent tools. Further, we assessed changes in microbial communities during microbe isolation to determine how the process can be improved.

**Results:**

Fourteen different variations of *C. jejuni* isolation procedures were applied to all 35 chicken fecal samples. These variations included using different enrichment broths (without enrichment or enrichment in Bolton or Preston broth), different ratios of sample-to-enrichment broth (1:10^1^, 1:10^2^, and 1:10^3^), and different selective agars (modified charcoal-cefoperazone-deoxycholate agar (mCCDA) or Preston agar). Enrichment during isolation of *C. jejuni* was evaluated on the basis of microbial diversity and taxonomic composition using metagenomics tools. The effect of selective media was evaluated using a combination of metagenomics and culture-dependent tools. Microbial diversity significantly decreased during the enrichment process, regardless of the type of enrichment broth, with the most significant decrease observed at a feces-to-broth ratio of 1:10^3^. Particularly, in 10^3^-Preston broth, the relative abundance of *Campylobacter* increased, while extended-spectrum beta-lactamase-producing *Escherichia coli*, which interfere with *Campylobacter* isolation, decreased. Metagenomics results were validated by quantitative PCR and culture-dependent analysis. Additionally, selective media affected the isolation results, although microbes with high relative abundance during enrichment were also frequently isolated using culture-dependent methods. Significantly more *C. jejuni* was isolated from mCCDA than from Preston agar enriched in 10^3^ Preston broth.

**Conclusions:**

Enrichment in Preston broth at a ratio of 1:10^3^ followed by spreading onto mCCDA was the most effective method for isolating *C. jejuni*. This is the first study to apply metagenomics to evaluate a method for isolating a targeted microbe, *C. jejuni*, from chicken feces, a source with high microbial contamination. Thus, metagenomics can be applied to improve methods for isolating bacteria that are difficult to separate.

**Electronic supplementary material:**

The online version of this article (10.1186/s40168-019-0680-z) contains supplementary material, which is available to authorized users.

## Background

*Campylobacter* spp., particularly *Campylobacter jejuni*, is a leading foodborne pathogen, worldwide [1]. Approximately nine million people in the European Union and 1.3 million people in the USA suffer from the *Campylobacter*-borne disease, and the number of infected people increases each year [2, 3]. A commensal organism that naturally colonizes and is asymptomatic in the gastrointestinal tract of poultry, particularly chickens, *C. jejuni* is a major cause of human campylobacteriosis [4, 5]. Symptoms of *C. jejuni* infections in humans include gastro-intestinal distress such as abdominal pain and diarrhea, as well as neurological issues including Guillain-Barre and Miller-Fisher syndrome [6]. Therefore, for the prevention and management of campylobacteriosis, isolation of *C. jejuni* from chicken should be prioritized. However, unlike other common foodborne pathogens, including pathogenic *Escherichia coli* and *Salmonella* spp., *C. jejuni* is difficult to isolate because of its specific growth conditions (i.e., requires a microaerophilic environment) [7]. The presence of viable but nonculturable (VBNC) *C. jejuni* and the tendency of *C. jejuni* to be masked by competing microbes, such as extended-spectrum beta-lactamase (ESBL)-producing *E. coli*, make it challenging to isolate *C. jejuni* from chickens [8, 9]. The most common methods for isolating *C. jejuni* from chicken meat are pre-enrichment (4–5 h, microaerophilic conditions, 37 °C) and enrichment (48 h, microaerophilic conditions, 42 °C) procedures in Bolton broth or Preston broth, followed by selection (48 h, microaerophilic conditions, 42 °C) on modified charcoal-cefoperazone-deoxycholate agar (mCCDA) or Preston agar. However, the prevalence of *C. jejuni* infections varies between studies, as there is no standard method, and outcomes differ depending on the type of enrichment broth and selective agar used [1, 10]. Additionally, most studies were performed using chicken meat. Because *Campylobacter* is a commensal flora of the gastrointestinal tract in poultry, it is important to isolate *Campylobacter* from the farm stage (feces of chicken). However, a method for isolating *C. jejuni* from chicken feces has not been established, and few studies have been performed [11].

The need for an effective method of isolating *C. jejuni* from chicken meat and feces has increased. Several studies were conducted comparing currently used methods for isolating *C. jejuni* and improving isolation by adding several antibiotics, such as polymyxin B and triclosan, to the enrichment broth or selective agar [7–9, 12]. However, most studies were carried out using culture-based techniques, which rely on the isolation results of a specific microbe, such as *Campylobacter* spp. and ESBL-producing *E. coli*. Changes in the overall microbial community and composition of specific microbes affecting the isolation of *C. jejuni* during isolation procedure have not been identified. Additionally, evaluation of the enrichment procedure using culture-based techniques, which greatly affects microbe isolation compared to selective procedures, is not possible [13]. Therefore, to accurately compare isolation methods of *C. jejuni*, studies based on metagenomics using next-generation sequencing are needed.

Currently, various next-generation sequencing (NGS) systems, such as Roche 454 pyrosequencing, Illumina Genome Analyzer (HiSeq, MiSeq), Applied Biosystems SOLiD System, Life Technologies Ion Torrent, and the PacBio RX system, are available in microbiological research fields [14]. Advances in these NGS technologies have enabled comprehensive analyses in microbiome analysis and whole-genome-sequencing analysis [15, 16]. However, most microbiological studies using these approaches have been conducted on the gut microbiome, which is closely linked to host health status [17–19]. Studies have been conducted to identify specific microorganisms based on sequence analysis but only for disease diagnosis [20]. However, disease diagnosis, alone, is not suitable for epidemiologic investigations or analyzing pathogenic factors, such as susceptibility to antibiotics, serotypes, subtypes, and virulence factors. These studies are possible only if the target microbes are isolated. Application of metagenomics approaches can improve the methods used to isolate specific microbes, particularly pathogens, by evaluating changes in microbial communities during pathogen isolation procedures.

The current study was carried out to evaluate the method for isolating *C. jejuni* from chicken feces through culture-independent sequence-based metagenomics tools and culture-dependent tools. First, the microbial community change, including alpha diversity, beta diversity, and taxonomic composition of chicken feces during the isolation procedures of *C. jejuni*, was analyzed for a total of seven procedures with different combinations of enrichment broth (enriched in Bolton or Preston broth or a no-enrichment process) and the ratio of sample-to-enrichment media (1:10^1^, 1:10^2^, and 1:10^3^). Second, the isolation results of *C. jejuni* and competing colonies in a total of 14 procedures, according to the combinations of the above seven procedures and type of selective media (mCCDA and Preston agar), were compared. Finally, the metagenomics results were validated by comparing the sequencing results to the culture-dependent results.

## Results

### *Campylobacte*r spp. and competing colonies isolated in each procedure determined using culture-dependent tools

**Fig. 1:**
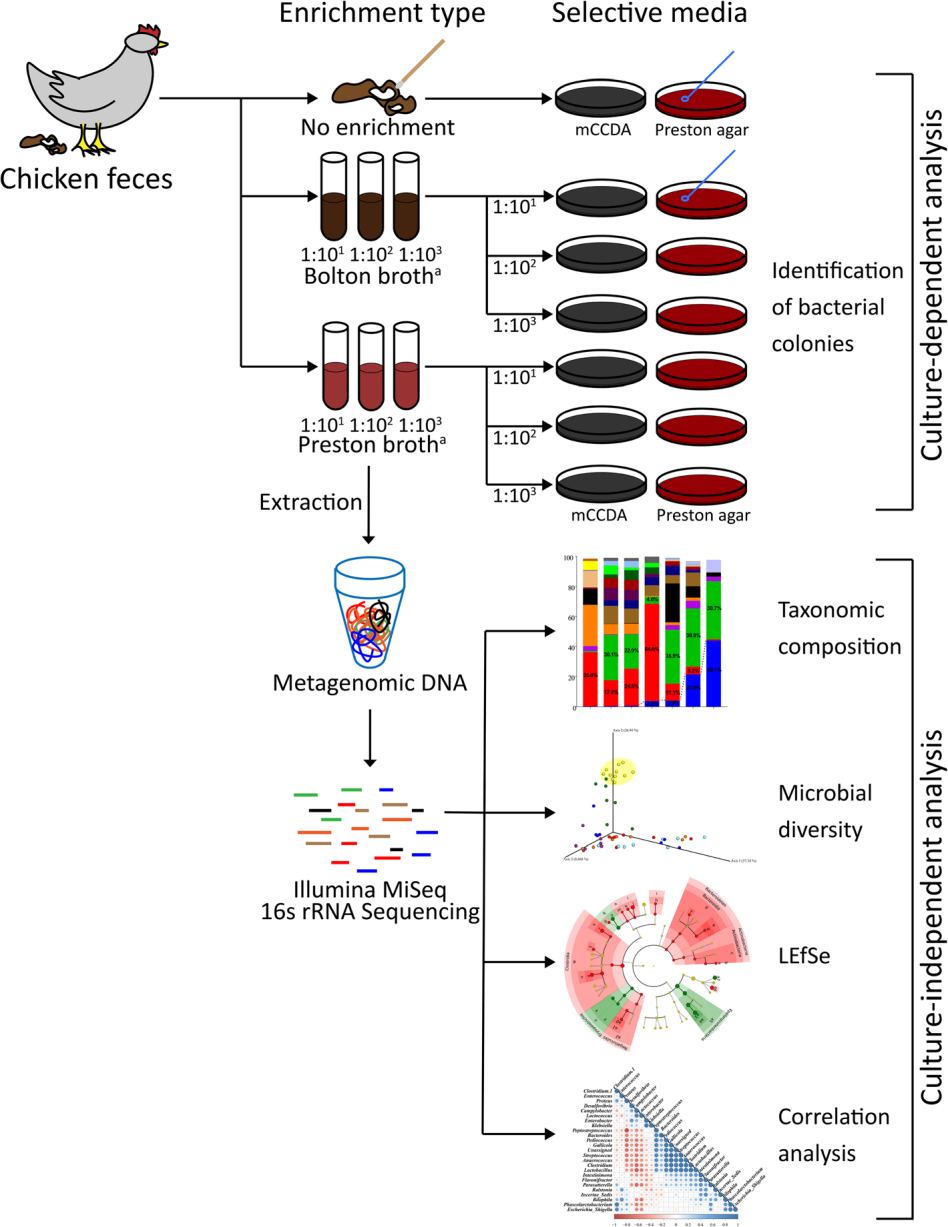
A schematic diagram of the current study. All chicken fecal samples were analyzed to compare isolation methods of *C. jejuni* using culture-independent sequence-based metagenomics and culture-dependent tools. Metagenomic analysis was performed to investigate the effects of seven enrichment procedures. Six procedures involved enrichment processes (Bolton and Preston broth at 1:10^1^, 1:10^2^, and 1:10^3^, respectively), and one procedure did not. In culture-dependent analysis, a total of 14 procedures were applied for all samples, which were combinations of different enrichment broths (without enrichment process or enriched in Bolton broth or Preston broth), ratio of sample-to-enrichment broth (1:10^1^, 1:10^2^, or 1:10^3^), and selective media (mCCDA or Preston agar). ^a^The ratio of fecal sample-to-enrichment broth

Comparison of the isolation results of *C. jejuni* according to each isolation method of *Campylobacter* revealed that among samples not subjected to the enrichment process, 45.7% (16/35) of *C. jejuni* was isolated from mCCDA and 57.1% (20/35) from Preston agar (Fig. 1). In samples enriched in Bolton broth, *C. jejuni* was not isolated, regardless of the ratio of sample-to-enrichment broth and types of selective agars. In Preston broth-mCCDA procedures, *C. jejuni* was not isolated from the 1:10^1^ ratio of sample-to-enrichment broth, while 31.4% (11/35) and 97.1% (34/35) of *C. jejuni* was isolated at ratios of 1:10^2^ and 1:10^3^, respectively. In the Preston broth-Preston agar procedures, *C. jejuni* was not isolated from the 1:10^1^ and 1:10^2^ ratios of sample-to-broth, while 5.7% (2/35) of *C. jejuni* was isolated from the 1:10^3^ ratio. The isolation rate of *C. jejuni* was significantly higher in the 10^3^-Preston broth-mCCDA procedure than in the other procedures (chi-square test, *p* < 0.05) and significantly higher in the 10^2^-Preston broth-mCCDA procedure than in the other procedures except for the 10^3^-Preston broth-mCCDA procedure (chi-square test, *p* < 0.05, Table 1).

**Table 1:** Culture-dependent isolation results of *Campylobacter jejuni* and competing microbes in each procedure

Enrichment type		No enrichment		Bolton broth						Preston broth					
Ratio		−		1:10^1^		1:10^2^		1:10^3^		1:10^1^		1:10^2^		1:10^3^	
Selective media		mCCDA	Preston agar	mCCDA	Preston agar	mCCDA	Preston agar	mCCDA	Preston agar	mCCDA	Preston agar	mCCDA	Preston agar	mCCDA	Preston agar
Target and competing microbes	*C. jejuni*	48.6% (17/35)	54.3% (19/35)	−	−	−	−	−	−	−	−	31.4% (11/35)^ac^	−	97.1% (34/35)^abc^	5.7% (2/35)
	ESBLs producing *E. coli*	48.6% (17/35)^a^	8.6% (3/35)	80.0% (28/35)^ac^	−	97.1% (34/35)^ac^	2.9% (1/35)	82.9% (29/35)^ac^	54.3% (19/35)^bc^	48.6% (17/35)^a^	−	42.9% (15/35)^a^	−	11.4% (4/35)^b^	−
	*P. mirabilis*	42.9% (15/35)	88.6% (31/35)^a^	45.7% (16/35)	94.3% (33/35)^a^	71.4% (24/35)	91.4% (32/35)	62.9% (22/35)^c^	82.9% (29/35)	65.7% (23/35)	94.3% (33/35)	54.3% (19/35)	80.0% (28/35)^a^	28.6% (10/35)^b^	97.1% (34/35) ^a^
	*Enterococcus* spp.	22.9% (8/35)^a^	2.9% (1/35)	−	−	−	−	−	−	20.0% (7/35)^ac^	2.9% (1/35)	31.4% (11/35)^ac^	2.9% (1/35)	17.1% (6/35)	8.6% (3/35)

A total of 14 procedures were applied for all 35 fecal samples, which were combinations of different enrichment type (without enrichment process or enriched in Bolton broth or Preston broth), ratio of sample-to-enrichment broth (1:10^1^, 1:10^2^, or 1:10^3^), and selective media (mCCDA or Preston agar)

^a^Significant differences according to selective media (enriched in the same enrichment broth and the same ratio of sample-to-broth)

^b^Significant differences according to the ratio of sample-to-broth (enriched in the same enrichment broth and same selective media)

^c^Significant differences according to the type of enrichment broth (enriched in the same ratio of sample-to-broth and the same type of selective media), −, negative result (0%)**.**

Additionally, ESBL-producing *E. coli* (all isolated *E. coli* were identified as ESBL-producing), *Proteus mirabilis*, and *Enterococcus* spp. were found in each procedure as colonies competing with *C. jejuni* (Table 1). For ESBL-producing *E. coli*, the isolation rate was significantly higher in mCCDA than in Preston agar (same enrichment broth type and ratio of sample-to-enrichment broth) and significantly higher in Bolton broth than in Preston broth (same ratio of sample-to-enrichment broth and selective media type) (chi-square test, *p* < 0.05, respectively). The isolation rate of ESBL-producing *E. coli* according to the ratio of sample-to-enrichment broth (in the same type of enrichment broth and selective agar) was significantly higher in 10^3^-Bolton broth/Preston agar and significantly lower in 10^3^-Preston broth/mCCDA (chi-square test, *p* < 0.05, respectively). For *P. mirabilis*, the isolation rate was significantly higher in Preston agar than in mCCDA (same enrichment broth type and the ratio of sample-to-enrichment broth) (chi-square test, *p* < 0.05). *Enterococcus* spp. was not isolated using the procedures enriched in Bolton broth, and the isolation rate was significantly higher in mCCDA than in Preston agar (same enrichment broth type and the ratio of sample-to-enrichment broth) (chi-square test, *p* < 0.05).

### Proportion of competing colonies and its relationship with isolation result of *C. jejuni* according to each procedure determined using culture-dependent tools

**Fig. 2:**
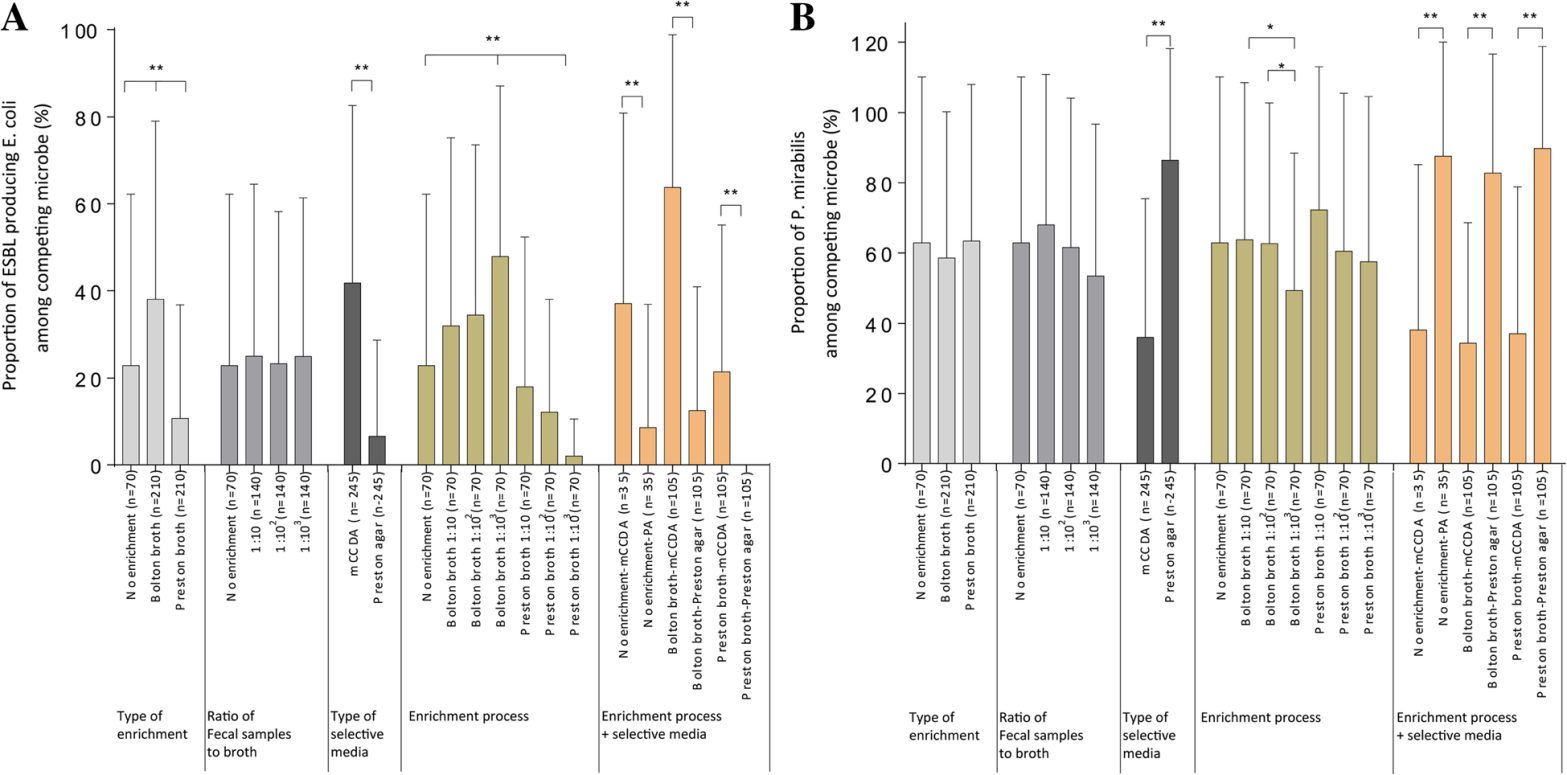
Proportion of competing microbes according to isolation procedures of *C. jejuni* based on culture-dependent tools. Proportion of **a** ESBL-producing *E. coli* and **b** *P. mirabilis* among competing microbes according to the isolation procedure of *C. jejuni* (mean ± SEM). Significance was determined by *t* test and one-way ANOVA. The proportion of ESBL-producing *E. coli* was significantly different according to the isolation procedure including the type of enrichment broth, selective media, and the combination of different enrichment broths, the ratio of sample-to-enrichment broth, and selective media. The proportion of *P. mirabilis* was significantly different according to the isolation procedure including the type of selective media and combination of different enrichment broths, the ratio of sample to enrichment broth, and selective agars. *p < 0.05, **p < 0.01

The ratio of the number of each colony to the total number of isolated colonies in the plate except for *C. jejuni* was used as a criterion for determining the proportion of competing colonies. Comparison of the proportion of each microbe according to enrichment type revealed that the proportions of ESBL-producing *E. coli* were significantly different (no enrichment process 22.9 ± 39.3%, Bolton broth 38.1 ± 40.9%, Preston broth 10.7 ± 26.1%, one-way ANOVA, *p* < 0.01), while *P. mirabilis* was not different (Fig. 2a, b). Comparison of the proportions of each microbe according to the selective media used showed that the proportion of ESBL-producing *E. coli* (mCCDA 41.8 ± 40.8%, Preston agar 6.9 ± 22.2%, *t* test, *p* < 0.01) and *P. mirabilis* (mCCDA 36.0 ± 39.5%, Preston agar 86.5 ± 31.7%, *t* test, *p* < 0.01) was significantly different. Comparison of the proportion of each microbe according to the ratio of sample-to-enrichment broth indicated that the proportion of ESBL-producing *E. coli* was significantly higher in 10^3^-Bolton broth, while the proportion of *P. mirabilis* was significantly lower in 10^3^-Bolton broth.

**Fig. 3:**
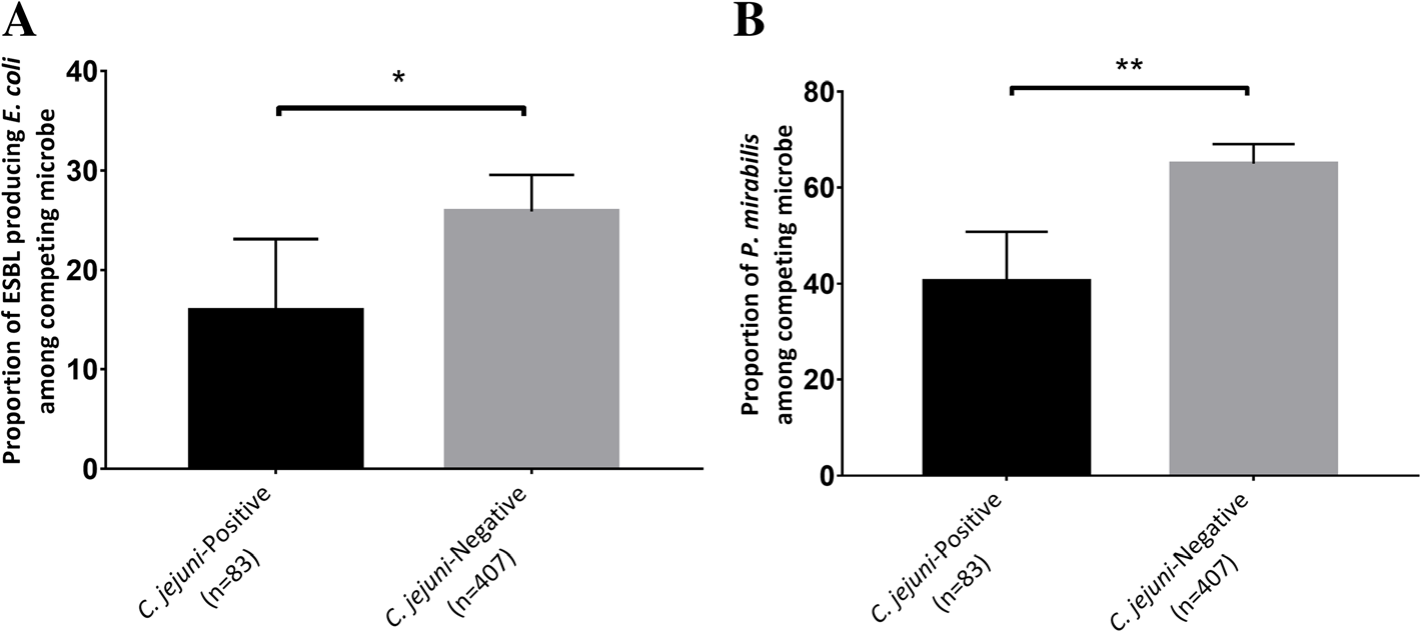
Evaluating the effect of competing flora on the isolation of *C. jejuni* using culture-dependent tools. The proportion of **a** ESBL-producing *E. coli* and **b** *P. mirabilis* according to the isolation results of *C. jejuni* (mean ± SEM). Significance was determined by *t* test. The proportion of ESBL-producing *E. coli* and *P. mirabilis* was significantly higher in *C. jejuni*-negative fecal samples. *p < 0.05, **p < 0.01

Additionally, the proportion of ESBL-producing *E. coli* and *P. mirabilis* significantly differed according to the isolation of *C. jejuni* in a total of 490 samples by applying 14 methods to each of 35 samples (Fig. 3a, b). In 83 *C. jejuni*-positive plates and 407 *C. jejuni*-negative plates, based on culture-dependent tools, the proportions of ESBL-producing *E. coli* were 15.9 ± 32.8% and 25.9 ± 22.2% ( *t* test, *p* < 0.05), respectively, while the proportions of *P. mirabilis* were 40.5 ± 46.7% and 65.0 ± 42.1%, respectively (*t* test, *p* < 0.01).

### Diversity of microbial communities in chicken feces of each enrichment process determined using metagenomics tools

**Fig. 4:**
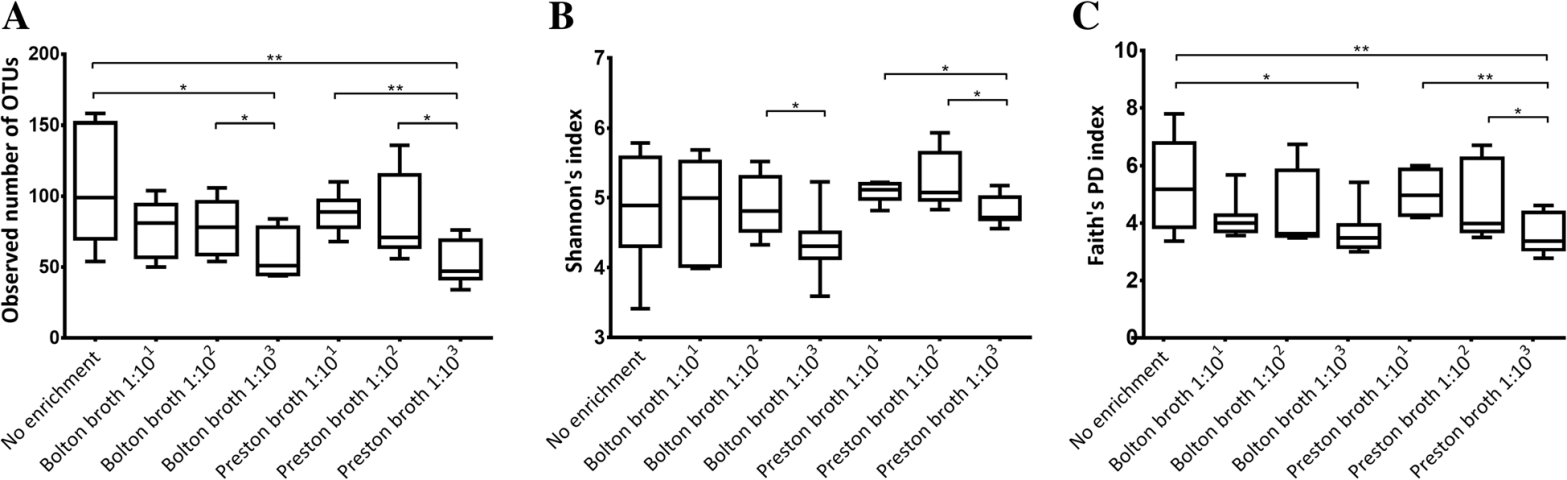
Alpha diversity of each seven enrichment procedures using metagenomics tools. **a** The number of observed operational taxonomic units (OTUs), **b** Shannon diversity index, and **c** Faith’s phylogenetic diversity in each procedure (min to max). Significance was determined by Mann-Whitney *U* test. The number of OTUs, microbial abundance and evenness, and microbial richness of fecal samples significantly decreased in the enrichment process as the ratio of sample-to- enrichment broth decreased. *p < 0.05, **p < 0.01

A total of 8,326,000 reads were obtained from 54 selected samples (all seven procedures for seven fecal samples and additional four fecal samples enriched in 10^3^-Preston broth and one additional fecal sample without enrichment) (Additional file 1: Table S1), of which 1,680,254 were valid after being filtered, de-replicated, and de-noised. As enrichment of the fecal samples progressed, the number of operational taxonomic units (OTUs) tended to decrease in the other processes (Fig. 4a). Additionally, the number of OTUs of the fecal samples in the non-enriched process was significantly higher than those in the 10^3^-Bolton (Mann-Whitney *U* test, *p* < 0.05) and Preston broth (Mann-Whitney *U* test, *p* < 0.01) processes. There was no significant difference in the number of OTUs between each enrichment broth type (same ratio of sample-to-enrichment broth). The Shannon index showed no significant difference between each enrichment broth type (same ratio of sample-to-enrichment broth). However, the Shannon index tended to decrease as the ratio of sample-to-enrichment broth decreased (Fig. 4b), particularly in 10^3^-Bolton and Preston broth (Mann-Whitney *U* test, *p* < 0.05 for each). Additionally, there was no difference between each type of enrichment broth in Faith’s phylogenetic diversity, while this value tended to decrease as the ratio of sample-to-enrichment broth decreased (Fig. 4c). Particularly, Faith’s phylogenetic diversity of fecal samples enriched in 10^3^-Preston broth was significantly decreased (Mann-Whitney *U* test, *p* < 0.05).

**Fig. 5:**
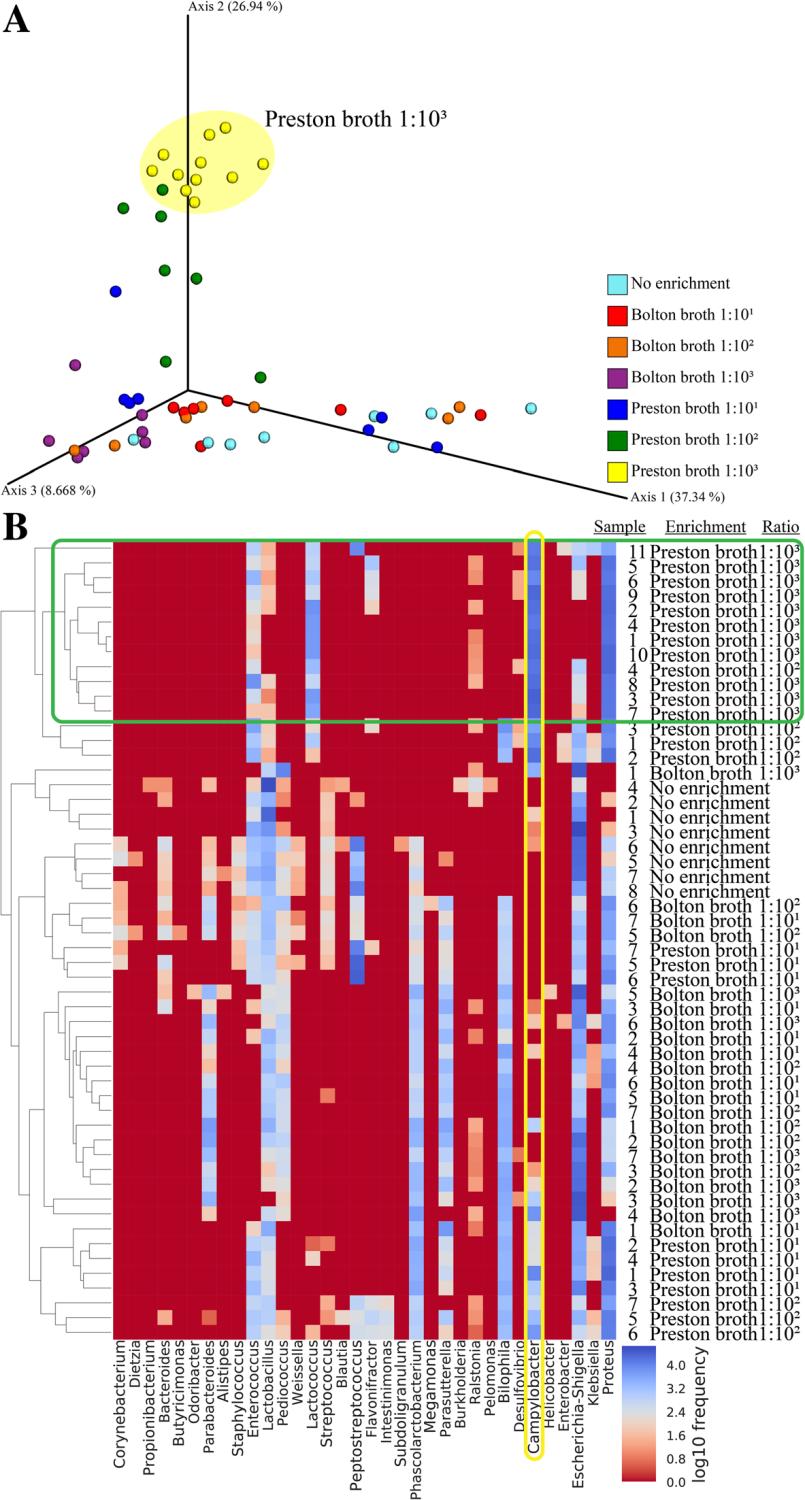
Similarity of the microbial community of fecal samples in each procedure. **a** Principal coordinates analysis (PCoA) plot of fecal samples (*n* = 54) in seven procedures based on weighted UniFrac distance metrics and **b** Heatmap of fecal samples (*n* = 54) in seven procedures. Fecal samples enriched in Preston broth at 1:10^3^ ratio were clustered together

The microbial communities in fecal samples in each of the seven enrichment processes were compared using a principal coordinates analysis (PCoA) plot based on a weighted UniFrac distance metric and heat map. The chicken feces that had not undergone enrichment, and fecal samples enriched in the Bolton broth process (regardless of the sample- to-broth ratio), were not clustered together. In 10^1^-/10^2^-Preston broth, the enriched fecal samples were not clustered together. However, fecal samples enriched in 10^3^-Preston broth were strongly clustered together (Fig. 5a). In the heat map, as well as the PCoA plot, fecal samples enriched in 10^3^-Preston broth were clustered together, unlike those enriched in Bolton broth or other ratios of sample-to-Preston broth (Fig. 5b). In permutational multivariate analysis (PERMANOVA) and analysis of similarities (ANOSIM) to detect differences between groups, the 10^3^-Preston broth process was significantly different from the other groups (*p* < 0.001).

### Composition of core microbial communities in feces of each enrichment process determined using metagenomics tools

**Fig. 6:**
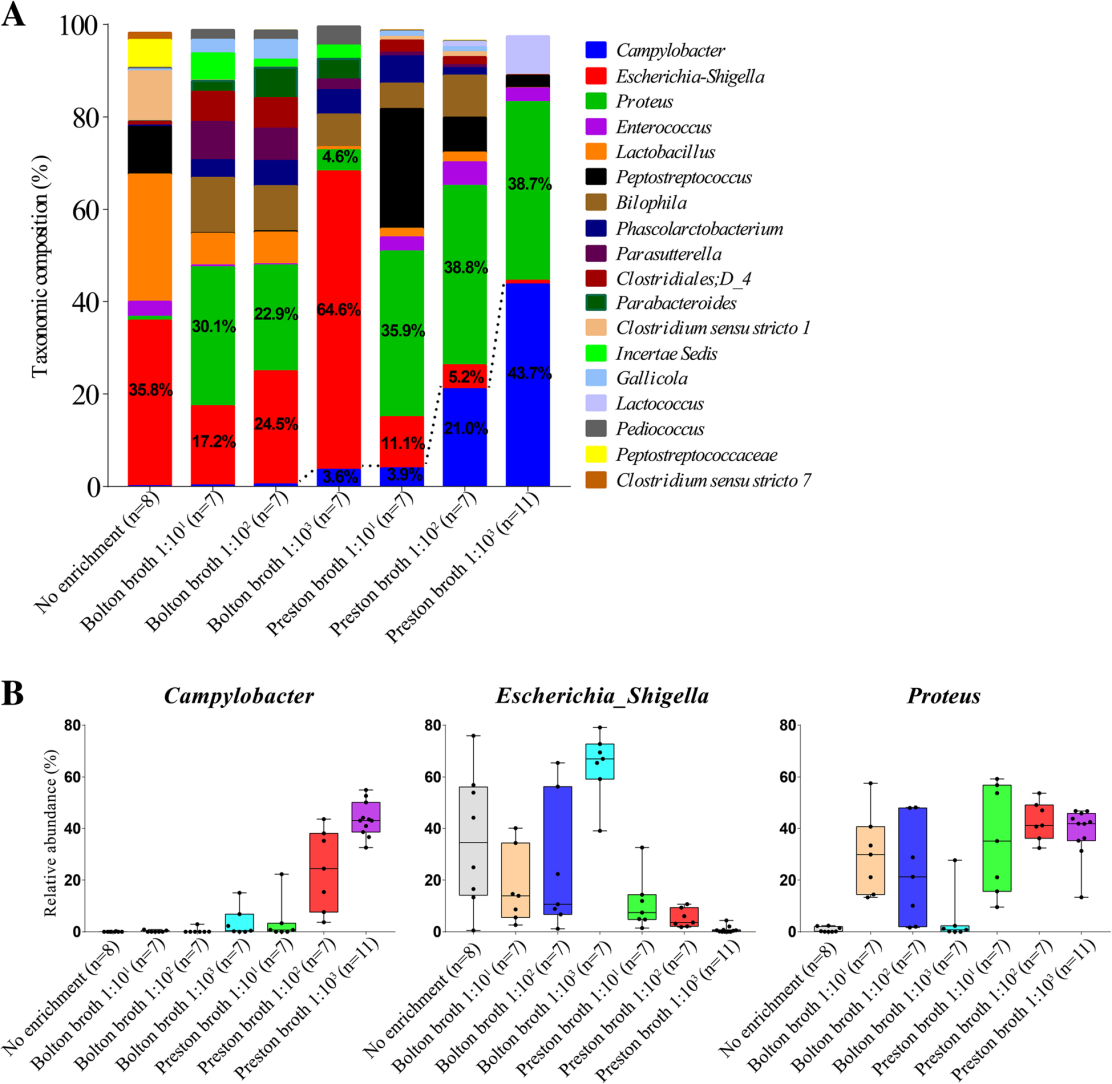
Microbial community of fecal samples in each procedure at the genus level. **a** Taxonomic composition and **b** relative abundance (min to max) of *Campylobacter*, *Escherichia*, and *Proteus* of fecal samples in each procedure. The relative abundance of *Campylobacter* in fecal samples in 10^3^–Preston broth was significantly higher than in other procedures. In the case of *Escherichia*-*Shigella*, the relative abundance was reduced, compared to feces, but was still present in large proportions in the process of 10^1^-/10^2^-Bolton broth enrichment. In the case of *Proteus*, the relative abundance increased during the enrichment process in both enrichment broths, at all ratios of sample-to- broth, with the exception of 10^3^-Bolton broth

The core taxonomic composition at the genus level (with an average of ≥ 1% in at least one process) is shown in Fig. 6a. At the genus level, 61 genera were identified; among these, 18 genera comprised the core microbiota in all processes and accounted for more than 98% of the total microbiota. In feces without the enrichment process, the predominant core microbiota included *Escherichia*-*Shigella* (35.8%), followed by *Lactobacillus* (27.6%), *Clostridium sensu stricto* 1 (10.8%), and *Peptostreptococcus* (10.3%). In the 10^1^-Bolton broth procedure, *Proteus* (30.1%), followed by *Escherichia*-*Shigella* (17.2%) and *Bilophila* (11.9%), were the predominant core microbiota; in the 10^2^-Bolton broth procedure, *Escherichia*-*Shigella* (24.5%) followed by *Proteus* (22.9%) were the predominant core microbiota; in the 10^3^-Bolton broth process, *Escherichia*-*Shigella* (64.6%) was the predominant core microbiota. In the 10^1^-Preston broth process, *Proteus* (35.9%), followed by *Peptostreptococcus* (25.9%), and *Escherichia*-*Shigella* (11.1%) were the predominant core microbiota; in the 10^2^-Preston broth process, *Proteus* (38.9%) followed by *Campylobacter* (21.0%) were the predominant core microbiota; in the 10^3^-Preston broth process, *Campylobacter* (43.7%), followed by *Proteus* (38.7%), were the predominant core microbiota.

### Relative abundance of *Campylobacter* and competing flora in feces of each enrichment process determined using metagenomics tools

The relative abundance of *Campylobacter* in feces that had not been subjected to the enrichment process and fecal samples enriched in Bolton broth was low, while that in fecal samples enriched in Preston broth tended to increase as the ratio of sample-to-broth decreased (Fig. 6b). In particular, the relative abundance of *Campylobacter* in fecal samples in 10^3^-Preston broth (43.7%) and 10^2^-Preston broth (21.0%) were significantly higher than in other procedures (Mann-Whitney *U* test, *p* < 0.01). The relative abundance of *Campylobacter* in fecal samples in 10^3^-Preston broth was significantly higher than that in 10^2^-Preston broth (Mann-Whitney *U* test, *p* < 0.05). The relative abundance of *Escherichia*-*Shigella* in fecal samples in Preston broth was reduced compared to in feces (Fig. 6b). The abundance of these bacteria tended to decrease as the ratio of sample-to-broth decreased during Preston broth enrichment, particularly in 10^3^-Preston broth (Mann-Whitney *U* test, *p* < 0.01). However, a substantial increase was observed in fecal samples enriched in 10^3^-Bolton broth (Mann-Whitney *U* test, *p* < 0.05) compared to other procedures. In the case of *Proteus*, the relative abundance was only 0.8% in feces not subjected to the enrichment process, while it was significantly increased in enrichment broth at all ratios of sample-to-broth (Mann-Whitney *U* test, *p* < 0.01), except for in 10^3^-Bolton broth (Fig. 6b). The abundance of *Proteus* in fecal samples in 10^3^-Bolton broth was significantly lower than in other procedures (Mann-Whitney *U* test, *p* < 0.01), except for feces that had not been subjected to the enrichment process. The results of linear discriminant analysis effect size (LEfSe) performed to compare 10^3^-Preston broth with other processes (no enrichment and 10^3^-Bolton broth) were also consistent (Additional file 3: Figure S1).

In correlation analysis, *Campylobacter* was negatively correlated with *Proteus* (Pearson correlation coefficient (*r*) − 0.39), and *Escherichia*-*Shigella* was also negatively correlated with *Proteus* (*r* − 0.82) in Bolton broth, regardless of the ratio of sample-to-broth (Additional file 4: Figure S2A). In Preston broth, regardless of the ratio of sample-to-broth, *Campylobacter* was negatively correlated with nearly all microbes including *Escherichia*-*Shigella* (*r* − 0.54) (Additional file 4: Figure S2B).

### Relationship between competing flora and *C. jejuni* determined using metagenomics tools and culture-dependent tools

**Fig. 7:**
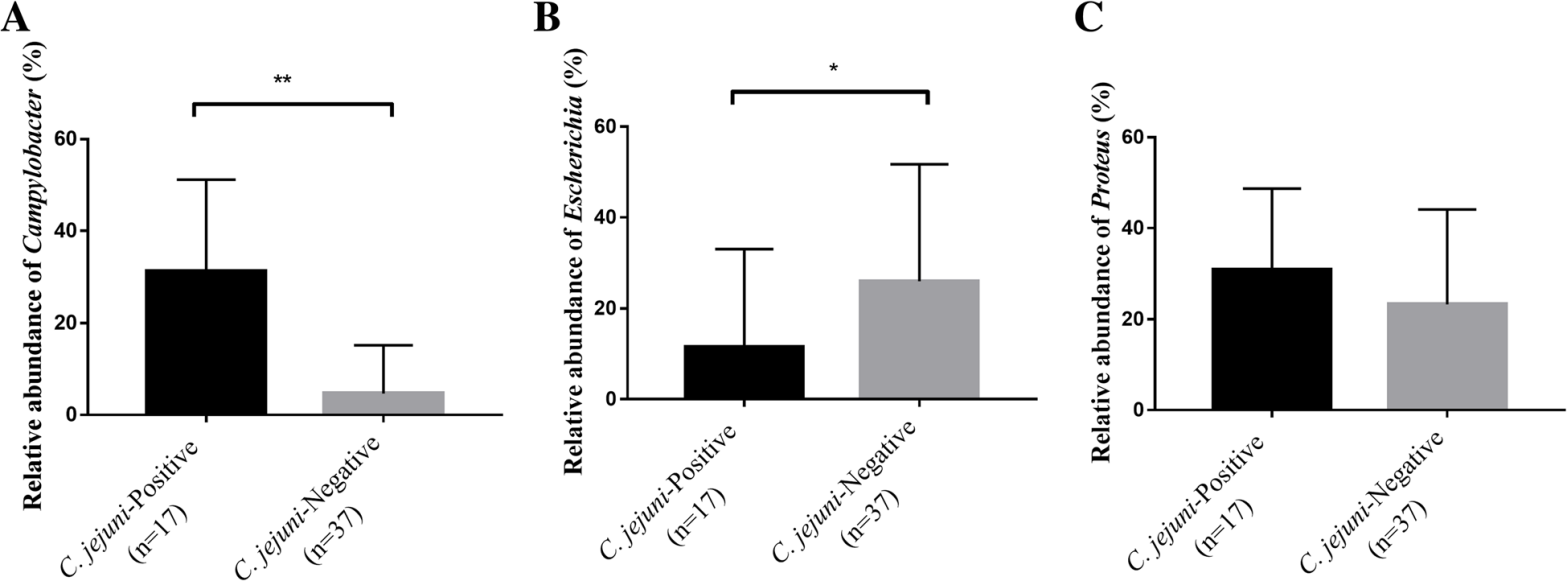
The relative abundance of each microbe according to the isolation results of *C. jejuni*. The relative abundance of **a** *Campylobacter*, **b** *Escherichia*, and **c** *Proteus* according to the isolation results of *C. jejuni* based on culture-dependent tools (mean ± SEM). A total of 54 samples analyzed by metagenomics tools were divided into two groups according to the culture results; 17 samples were positive for *C. jejuni*, and 37 were negative. Significance was determined by *t* test. The relative abundance of *Campylobacter* was significantly higher in the fecal sample from which *C. jejuni* was isolated, while the relative abundance of *Escherichia* was significantly lower. There was no difference in the relative abundance of *Proteus* in the fecal sample according to isolation result of *C. jejuni*. *p < 0.05, **p < 0.01

A total of 54 samples analyzed by metagenomics tools were divided into two groups according to the culture results; 17 samples were positive for *C. jejuni*, and 37 were negative. The relative abundance of *Campylobacter* was significantly higher in fecal samples from which *C. jejuni* was detected based on the culture result (*t* test, *p* < 0.01, Fig. 7a). However, the relative abundance of *Escherichia* was significantly lower in fecal samples from which *C. jejuni* was detected based on the culture result (*t* test, *p* < 0.05, Fig. 7b). For *Proteus*, there was no difference in the relative abundance in the fecal sample according to isolation result of *C. jejuni* based on the culture result (Fig. 7c).

### Validation of metagenomics data using quantitative PCR

**Fig. 8:**
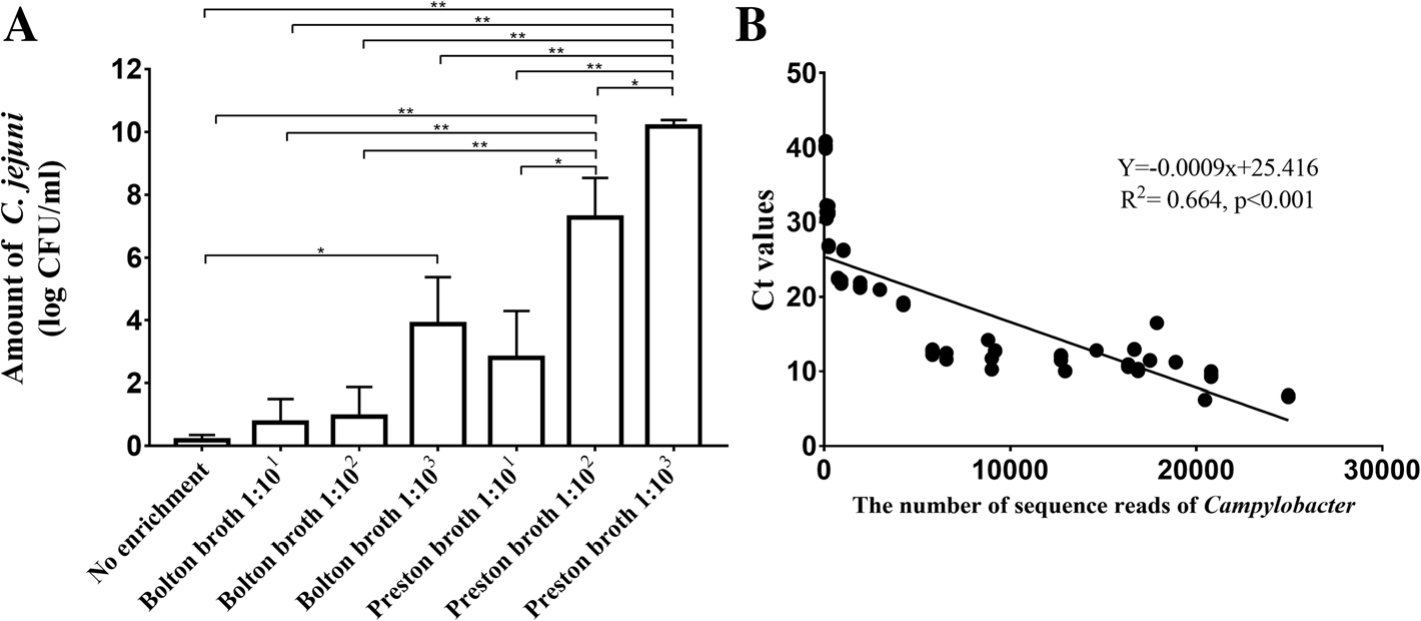
Quantitative PCR of *C. jejuni* applied to all 54 samples. **a** Log CFU/ml of *C. jejuni* in each procedure (mean ± SEM) inferred from the standard curve and **b** correlation of metagenomics data and quantitative polymerase chain reaction (qPCR) results. Significance was determined by Mann-Whitney *U* test. The amount of *C. jejuni* was significantly higher in fecal samples in 10^3^-Preston broth, 1.0 × 10^10.2 ± 0.2^, followed by 10^2^-Preston broth (1.0 × 10^7.3 ± 1.3^) and 10^3^-Bolton broth (1.0 × 10^3.9 ± 1.5^). In addition, the cycle threshold (Ct) values from qPCR results and the number of *Campylobacter* reads from metagenomics results had a high correlation. *p < 0.05, **p < 0.01

Quantitative PCR (qPCR) of *C. jejuni* was conducted on all 54 samples to validate the metagenomics data and estimate the levels of *C. jejuni* in each procedure. First, qPCR was performed using standard strains (NCTC 11168 and ATCC 33560) to confirm the correlation between colony-forming units (CFU) and cycle threshold (Ct) values of *C. jejuni* (*y* = − 3.3631x + 44.547, *R*^2^ = 0.9118, *p* < 0.001) (Additional file 5: Figure S3). Next, qPCR was conducted on the DNA used in the current study. The log CFU/ml of *C. jejuni* in each sample was inferred through the standard curve: 0.2 ± 0.2 (no enrichment, mean ± SEM), 0.7 ± 0.7 (10^1^-Bolton broth), 0.9 ± 0.9 (10^2^-Bolton broth), 3.9 ± 1.5 (10^3^-Bolton broth), 2.8 ± 1.5 (10^1^-Preston broth), 7.3 ± 1.3 (10^2^-Preston broth), and 10.2 ± 0.2 (10^3^-Preston broth) (Fig. 8a). The amount of *C. jejuni* was significantly higher in fecal samples in 10^3^-Preston broth than in the other procedures (Mann-Whitney *U* test, *p* < 0.05). Additionally, the amount of *C. jejuni* was significantly higher in fecal samples in 10^2^-Preston broth than in the other procedures (Mann-Whitney *U* test, *p* < 0.05), except for the 10^3^-Preston/Bolton broth. Furthermore, the correlation between the Ct values from the qPCR results and number of *Campylobacter* reads from metagenomics results was examined (*y* = − 0.0009x + 25.416, *R*^2^ = 0.664, *p* < 0.001, Fig. 8b).

## Discussion

This is the first study to apply culture-independent sequence-based metagenomics to evaluate *C. jejuni* isolation methods in chicken feces. Isolation of *Campylobacter spp.* is difficult because of the specific growth conditions of the bacteria, presence of VBNC bacteria, and masking by competing flora on selective media. Therefore, culture-independent diagnostic tests, which show high sensitivity and rapid diagnosis, were recently applied to detect *Campylobacter*-infections, particularly by the Centers for Disease Control Foodborne Diseases Active Surveillance Network [21]. However, applying culture-independent diagnostic tests alone without obtaining isolates from culture-dependent methods makes further analyses, such as epidemiological investigation and characterization of strains, impossible. Given this, effective methods for isolating *C. jejuni* are needed. Numerous studies have been conducted to evaluate and improve the methods of isolating *C. jejuni* from various food sources using culture-dependent tools [7, 8, 22]. Although food sources are the main cause of human campylobacteriosis, isolation of *C. jejuni* at the farm level is primarily necessary for managing campylobacteriosis. However, most studies have been carried out using chicken meat with low microbial contamination rather than chicken feces, which is a high microbial contamination source [23, 24]. Therefore, to isolate *Campylobacter* from feces, a specific method, which differs from that used for food, should be applied. Here, the methods for isolating *C. jejuni* from chicken feces were evaluated using metagenomics tools and culture-dependent tools.

Based on metagenomics analysis, the current study indicated that feces undergoing an enrichment process in Preston broth at a 1:10^3^ ratio of feces-to-broth were the most effective for isolating *C. jejuni*. In metagenomics applications to evaluate the isolation method of *Campylobacter*, microbial diversity and taxonomic composition were calculated. First, the effect of the enrichment process during the isolation of *C. jejuni* was assessed based on microbial diversity. Based on the alpha diversity, including the number of OTUs, Shannon diversity index which measures species abundance and evenness, and Faith’s phylogenetic diversity index which measures biodiversity, the diversity of the microbial community in the feces was decreased during enrichment compared to fecal samples that were not enriched [25, 26]. The microbial diversity decreased to a greater extent as the ratio of feces-to-broth decreased, particularly when the level of enrichment broth was high compared to the fecal samples. However, at the same ratio of fecal samples-to-each enrichment broth, there was no significant difference in microbial diversity. This suggests that the ratio of feces-to-broth affects microbial diversity, regardless of the type of enrichment broth. This was because during the enrichment process for *C. jejuni*, only the target microbe selectively grew, and other microbes were inhibited by antibiotics and specific enrichment conditions (microaerophilic conditions and a high incubation temperature of 42 °C). Additionally, the difference in microbial diversity according to the ratio of fecal samples-to-broth may be related to differences in the relative amounts of antibiotics-to-fecal samples. A greater relative amount of antibiotics-to-fecal samples led to greater inhibitory effects of antibiotics on the fecal sample.

Second, the effect of the enrichment process was evaluated based on taxonomic composition. In non-enriched feces, *Escherichia*-*Shigella* and *Lactobacillus* were the most abundant genera. This is similar to results of previous studies showing that *Lactobacillus* (14.9%) and *Escherichia-Shigella* (9.0%) are the most abundant bacteria in feces from healthy chickens [27]. However, the original taxonomic composition in the feces changed dramatically during the enrichment process. The relative abundance of *Campylobacter* (identified as *C. jejuni* at the species level using metagenomics and culture-based tools) was significantly higher in Preston broth, especially in 10^2^- and 10^3^-Preston broth, than in Bolton broth. In Preston broth, the relative abundance of *Campylobacter* increased as the ratio of feces-to-broth decreased from 1:10^1^ to 1:10^3^. Furthermore, qPCR was performed to validate the metagenomics data, which varied depending on the type of sequencer used, type of database, and DNA extraction method [14, 28, 29]. The number of reads of *C. jejuni* from metagenomics data and qPCR results of *C. jejuni* was highly correlated, suggesting that the metagenomics data in this study was reliable, and the amount of *C. jejuni* in each enrichment process was deduced. The amount of *C. jejuni* was high in fecal samples in 10^3^-Preston broth at 1.0 × 10^10.2 ± 0.2^. This was similar to that for *C. jejuni* in brain heart infusion broth at 48 h (1.0 × 10^8^–1.0 × 10^9^) [30]. The negative qPCR results for *C. jejuni* may be due to DNA contamination from other bacteria, or that transcription was below the detection limit.

Differences in the levels of *C. jejuni*, according to enrichment procedures, were observed because of competing flora, particularly ESBL-producing *E. coli*, which were influenced by the type of antibiotics in each enrichment broth and by the relative amount of antibiotics-to-feces in each ratio of broth. In general, the amount of background flora affects the isolation result of *C. jejuni*, often producing false-negative results [31]. Comparison of the two enrichment broth types revealed that polymyxin B effectively controlled ESBL-producing *E. coli*; this antibiotic is present in Preston broth but not in Bolton broth [9]. Recent studies also showed that ESBL-producing *E. coli* is poorly resistant to the antibiotics cefoperazone, vancomycin, and trimethoprim, which are present in Bolton broth [8]. Additionally, the growth of *C. jejuni* in co-cultures of *C. jejuni* and ESBL-producing *E.coli* was significantly lower than in the single culture of *C. jejuni*, indicating that ESBL-producing *E. coli* acts as a competitive inhibitor leading to a reduction of *C. jejuni* levels [32]. In our study, the relative abundance of *Escherichia* (identified as ESBL-producing *E. coli* using culture-based tools), which was highest in non-enriched feces, showed a more substantial decrease in Preston broth than in Bolton broth at the same ratio of fecal samples-to-enrichment broth and tended to decrease as the ratio of feces-to-Preston broth decreased. *Campylobacter* was negatively correlated with *Escherichia* when fecal samples were enriched in Preston broth. Therefore, in the enrichment process of *Campylobacter*, Preston broth more effectively inhibited ESBL-producing *E. coli* compared to Bolton broth. Furthermore, a comparison of the ratio of fecal samples-to-enrichment broth of 1:10^1^ to 1:10^3^ in Preston broth revealed that the differences depended on the amount of antibiotics used. The ratio of sample-to-enrichment broth of 1:10^1^, which is the most commonly used ratio for isolating *Campylobacter* from food sources, did not effectively enrich target microbes in the fecal samples. This means that antibiotics in the enrichment broth may not work effectively in the enrichment process unless the ratio of feces-to-enrichment broth is optimized. Therefore, the relative amount of antibiotics should be high in order to inhibit competing microbes when isolating *C. jejuni* from feces with higher microbial contamination than in food. Moreover, fecal samples enriched in 10^3^-Preston broth were strongly clustered together in the PCoA plot and heat map, while fecal samples in the other procedures were not clustered with each other. These results suggest that 10^3^-Preston broth has the most constant effect on fecal samples during the enrichment process, including decreasing microbial diversity and changing the microbial composition.

*P*. *mirabilis* was a major competing microbe observed during the process of isolating *Campylobacter* in previous studies [33]. The current study also showed that the relative abundance of *Proteus* (identified as *P. mirabilis* at the species level using metagenomics tools and culture-based tools) in the enrichment process increased, compared to fecal samples that were not enriched. This may be because of their high ability to differentiate and survive [34, 35]. However, the relative abundance of *Proteus* did not affect the relative abundance of *Campylobacter*, unlike ESBL-producing *E. coli*. Instead, the relative abundance of *Proteus* was high in Preston broth at 1:10^2^ and 1:10^3^ ratios, but low in Bolton broth at a 1:10^3^ ratio. This was most likely due to resistance to polymyxin B in Preston broth, but susceptibility to vancomycin in Bolton broth [36]. We also predict that competition occurred between *Proteus* and ESBL-producing *E. coli* based on the negative correlation between these two microbes in Bolton broth, although further studies are needed to confirm this.

Analysis of *C. jejuni* isolation procedures using metagenomics was confirmed using culture-dependent tools. *Campylobacter*, *Escherichia-Shigella*, and *Proteus* sequence reads based on metagenomics tools matched those of isolates obtained by culture-dependent tools. *Campylobacter* and *Proteus* were identified as *C. jejuni* and *P. mirabilis* at the species level, respectively, and only *C. jejuni* and *P. mirabilis*, without other species, were isolated based on culture-dependent tools. For *Escherichia-Shigella* at the genus level, although species-level identification was not possible, only *E. coli* was isolated, all of which were identified as ESBL-producing *E. coli* using culture-dependent methods. The microbes that, using metagenomics, were identified to be present at a relatively higher abundance in each enrichment process were also frequently isolated using culture-dependent methods, regardless of the type of selective media used. The isolation rates of *C. jejuni*, ESBL-producing *E. coli*, and *P. mirabilis* were significantly higher in enriched fecal samples, with a higher relative abundance of all three. Additionally, the inhibitory effect of competing flora in the isolation of *C. jejuni*, which was determined using metagenomics tools, was confirmed using culture-dependent tools; the proportion of ESBL-producing *E. coli* and *P. mirabilis* was significantly higher in *C. jejuni*-negative fecal samples than in *C. jejuni*-positive fecal samples. The consistency between the culture-based results and metagenomics results suggests that our evaluation of the isolation method using metagenomics tools was reliable.

The effect of selective media was evaluated based on the use of culture-dependent tools. Using metagenomics alone, only the effects of the enrichment process on the isolation of *Campylobacter* can be evaluated; however, using a combination of metagenomics and culture-dependent tools enabled evaluation of the enrichment broth and selective media in isolating *Campylobacter*. Generally, the enrichment step had a greater effect on the isolation of microbes than did the selective media [13]. However, we found that selective media significantly affected target microbe isolation as well. Even if the fecal sample was subjected to the same enrichment process (same type of enrichment broth and ratio of feces-to-enrichment broth), the isolation rate and proportion of microbes differed depending on the selective media used. ESBL-producing *E. coli* was isolated from mCCDA significantly more frequently than from Preston agar, while *P. mirabilis* isolation was greater from Preston agar than from mCCDA. This was most likely due to the antibiotics present in the selective media. The antibiotics in mCCDA were similar those in Bolton broth, while the antibiotics in Preston agar were consistent with those in Preston broth. Because of this, Preston agar and broth showed similar inhibitory effects on ESBLs, while mCCDA inhibited *Proteus* as in Bolton broth. Particularly, Preston agar, which does not inhibit *P. mirabilis*, made it difficult to isolate *C. jejuni* because of the swarming differentiation characteristics of *P. mirabilis*, which colonized the entire plate surface causing a masking phenomenon [37]. Additionally, in the enrichment process in 10^2^- and 10^3^-Preston broth, while the same type of enrichment broth was used, the isolation rate of *C. jejuni* was significantly higher in Preston agar than in mCCDA. This may be due to a cross-selective inhibition effect of antibiotics in enrichment broth and selective media. The relative abundance of ESBL-producing *E. coli* decreased during the enrichment process in Preston broth, and *P. mirabilis* was inhibited in mCCDA, facilitating the isolation of *C. jejuni*.

## Conclusions

This is the first study to apply culture-independent sequence-based metagenomics and culture-dependent tools to evaluate *C. jejuni* isolation methods from feces, a high-level microbial matrix. The application of metagenomics revealed changes in the microbial community during the microbial isolation procedure, which cannot be detected using previous culture-based analyses. Particularly, changes in the target microbe and competing flora during the enrichment process, and their correlation, were observed. Our results suggest that (1) Preston broth is more effective for isolating *C. jejuni* from chicken feces compared to Bolton broth, which is widely used to isolate this bacteria from chicken meat; (2) the effectiveness of the enrichment process depends on the ratio of samples to enrichment broth, even in enrichment broth containing effective antibiotics; (3) when isolating microorganisms, although the type and ratio of enrichment broth should be considered, the type of selective agars is also essential. Taken together, we optimized the method for isolating *C. jejuni* from chicken feces, enriched in Preston broth at a ratio of 1:10^3^ (sample-to-broth) followed by spreading onto mCCDA. This procedure effectively enriched *C. jejuni* and controlled competing flora, particularly ESBL-producing *E. coli* and *P. mirabilis.* This isolation method aids in obtaining *Campylobacter* isolates effectively, enabling further analyses, which include epidemiological investigation and molecular characterization of strains. This study provides a new perspective and possibilities for applying metagenomics in microbiological research, such as improving diagnostic methods for fastidious bacteria that are difficult to isolate.

## Methods

### Sampling of chicken feces and applying different isolation methods of *C. jejuni*

In December 2017, 35 fecal samples from the gastrointestinal tract of chickens at a slaughterhouse were collected. All fecal samples were analyzed to compare isolation methods of *C. jejuni* (Fig. 1 and Additional file 1: Table S1). A total of 14 procedures were applied for all samples, which were combinations of different enrichment broths (without enrichment process or enriched in Bolton broth or Preston broth), ratio of sample-to-enrichment broth (1:10^1^, 1:10^2^, or 1:10^3^), and selective media (mCCDA or Preston agar). First, one loop (10 μL) of all 35 fecal samples was directly spread onto two different types of selective media, mCCDA (Oxoid Ltd., Hampshire, UK) with a CCDA selective supplement (Oxoid Ltd.) and Preston agar (Oxoid Ltd.) with a Preston Campylobacter selective supplement (Oxoid Ltd.) and lysed horse blood (Oxoid Ltd.) without enrichment. The selective media were microaerobically incubated at 42 °C for 48 h. Second, all 35 fecal samples were enriched in Bolton broth (Oxoid Ltd.) with a Bolton broth selective supplement (Oxoid Ltd.) at different ratios of sample-to-enrichment broth (1:10, 1:10^2^, and 1:10^3^, respectively). Next, the enrichment broths were micro-aerobically incubated at 42 °C for 48 h, followed by plating onto mCCDA and Preston agar at 42 °C for 48 h under microaerobic conditions. Third, all 35 fecal samples were enriched in Preston broth (Oxoid Ltd.) with a Preston broth selective supplement (Oxoid Ltd, Hampshire, UK) at different ratios of sample-to-enrichment broth (1:10, 1:10^2^, and 1:10^3^, respectively). The enrichment broths were microaerobically incubated at 42 °C for 48 h, followed by plating onto mCCDA and Preston agar at 42 °C for 48 h microaerobically.

### Identification of bacterial colonies obtained from culture-dependent tools

In 14 different procedures, at least four colonies suspected as *Campylobacter* spp. were transferred to Müller-Hinton agar (MHA, Oxoid Ltd.) and microaerobically incubated at 42 °C for 48 h. Next, *C. jejuni* was confirmed by PCR for each colony (Additional file 2: Table S2) [38]. For colonies that did not show typical morphology of *Campylobacter* spp., an average of 2.47 (range 1–9) colonies with different shapes was transferred to MHA, according to the proportion of colonies constituting the selective agar. The agars were incubated at 42 °C for 48 h under microaerobic conditions. Colonies suspected to be *Escherichia coli* and *Enterococcus faecium/faecalis* were confirmed by PCR (Additional file 2: Table 2) [39, 40]. For colonies identified as *E. coli*, an antibiotic resistance test was performed according to the Clinical and Laboratory Standards Institute guidelines to identify ESBL-producing *E. coli*. Additionally, colonies not confirmed by PCR (*C. jejuni*, *E. coli*, *E. faecium*, *E. faecalis*) were identified by 16 s rRNA sequencing using the universal 16S rRNA primers 518F and 805R [41].

### DNA extraction and Illumina MiSeq sequencing

Microbial community analysis of feces and enriched fecal samples was performed to investigate the effects of seven enrichment procedures using different types of enrichment broth and ratios of sample-to-enrichment broth. Six procedures involved enrichment processes (Bolton and Preston broth at 1:10^1^, 1:10^2^, and 1:10^3^, respectively), and one procedure did not. At least seven samples per each procedure were selected according to the selection criteria (isolation result of *C. jejuni* based on the culture-dependent tool), and a total of 54 samples (8 feces and 46 enriched fecal samples) were examined in microbial community analysis (Additional file 1: Table S1). Metagenomic DNA was extracted using a FastDNA SPIN extraction kit (MP Biomedicals, Santa Ana, CA, USA) according to the manufacturer’s instructions. DNA amounts and quality were quantified using PicoGreen (Thermo Scientific, Waltham, MA, USA) and a Nanodrop spectrometer (Thermo Scientific). Bacterial DNA amplification was carried out, targeting the V3–V4 regions of the 16S rRNA gene (Additional file 2: Table S2) [42]. Next, to add multiplexing indices and the sequencing adapter, a subsequent amplification was performed. The amplified products were pooled and normalized using PicoGreen. Sequencing was conducted at Macrogen, Inc. (Seoul, Korea), using an Illumina MiSeq (Illumina, San Diego, CA, USA).

### Processing of sequencing analysis

Bioinformatic analysis was performed using QIIME2 software (version 2018.2) pipeline [43]. Raw sequence reads were filtered, de-replicated, and de-noised using DADA2’s recommended parameters [44]. Phylogenetic diversity analysis was performed using MAFFT, and a phylogenetic tree was created using the midpoint rooting method with the FastTree plugin [45, 46]. Alpha diversity was measured in QIIME2 with a subsampling depth of 12,001 sequences, and beta diversity was measured in QIIME2 based on the weighted UniFrac distance [47, 48]. Taxonomy assignments were conducted using the SILVA database version 119 [29]. Pearson’s correlation coefficient was calculated in MicrobiomeAnalyst, and a correlation matrix plot was generated using R version 3.5.0 [49]. To identify the bacterial taxa that are differentially abundant in each group’s microbial community, LEfSe analysis was performed using the online Galaxy interface (http://huttenhower.sph.harvard.edu/galaxy/), and the logarithmic linear discriminant analysis score cutoff was set to 2.0.

### Quantitative PCR of *C. jejuni*

Quantitative PCR of *C. jejuni* was performed using DNA extracted from ATTC 33560 and NCTC 11168 (DNA concentration was 0.0000237–237 ng/μL) and 54 DNA samples subjected to microbial community analysis. *hipO* was selected as a target gene for distinguishing *C. jejuni* from other microbes. Primers and probes for qPCR were used with slight modifications from previous studies (Additional file 1: Table S1) [50]. All reactions were performed in a total volume of 25 μL containing 5 μL of DNA, 2.5 μL of primers (0.8 μM of each primer), 0.5 μL of probe (0.2 μM), 12.5 μL of 2x TaqMan Universal Master Mix II (Life Technologies, Carlsbad, CA, USA), and 4.5 μL of distilled water. The amplification conditions were 95 °C for 10 min, followed by 45 cycles at 95 °C for 15 s and 58 °C for 1 min. All reactions were run in triplicate using an ABI PRISM 7500 fast real-time PCR system (Life Technologies).

### Statistical analysis

Chi-squared test was performed to compare the isolation rates of *C. jejuni* and competing colonies between procedures. One-way ANOVA and unpaired *t* test were performed to compare the proportion of competing colonies grown on the plates, for each isolation procedure. In addition, the Mann-Whitney *U* test was performed to compare microbial communities, including the microbial diversity and taxonomic composition of bacteria in fecal samples, according to enrichment procedures. All statistical analyses were performed in SPSS version 22.0 (SPSS, Inc., Chicago, IL, USA) and a *p* value of < 0.05 was accepted to indicate statistical significance. Additionally, regression analysis was performed using SPSS to correlate and validate the microbiota data. PERMANOVA and ANOSIM were performed to evaluate similarity among groups (based on weighted UniFrac distance).

## Additional files

Additional file 1: **Table S1.** Information of samples and isolation results of *C. jejuni*. CJ: positive for *C. jejuni* in culture-based results, Blank: negative for *C. jejuni* in culture-based results. Gray color: samples used for microbial community analysis. A total of 54 samples (at least seven samples per process) were used for microbial community analysis.

Additional file 2: **Table S2.** Primer list for polymerase chain reaction (PCR), quantitative PCR, and bacterial DNA amplification in this study. (DOCX 16 kb)

Additional file 3: **Figure S1.** Bacterial taxa that are differentially abundant in the microbial community of fecal samples in each procedure. A) Linear discriminant analysis effect size (LEfSe) and B) taxonomic cladogram between fecal samples enriched in Preston broth at the 1:10^3^ ratio and not undergoing the enrichment process. C) LEfSe and D) taxonomic cladogram between fecal samples enriched in Preston broth and Bolton broth at the 1:10^3^ ratio. The logarithmic linear discriminant analysis score cutoff was set to 2.0. The relative abundance of *Campylobacter* in fecal samples in 10^3^-Preston broth was significantly higher than in other procedures, while the relative abundance of *Escherichia*-*Shigella* was significantly lower than in other procedures. (TIF 935 kb)

Additional file 4: **Figure S2.** Relationship between microorganisms in microbial community of fecal samples. Correlation plot in A) Bolton broth and B) Preston broth regardless of the ratio of sample- to-enrichment broth. *Campylobacter* was negatively correlated with *Proteus* in Bolton broth, while *Campylobacter* was negatively correlated with *Escherichia*-*Shigella* in Preston broth. (TIF 711 kb)

Additional file 5: **Figure S3.** The correlation between colony forming-units and cycle threshold (Ct) values of *C. jejuni* standard strains (NCTC 11168 and ATCC 33560). (TIF 155 kb)

## Abbreviations

ANOSIM: Analysis of similarities
CFU: Colony-forming units
Ct: Cycle threshold
ESBL: Extended-spectrum beta-lactamase
LEfSe: Linear discriminant analysis effect size
mCCDA: Modified charcoal-cefoperazone-deoxycholate agar
OTU: Operational taxonomic units
PCoA: Principal coordinates analysis
PCR: Polymerase chain reaction
PERMANOVA: Permutational multivariate analysis
qPCR: Quantitative PCR
*r*: Pearson correlation coefficient
VBNC: Viable but nonculturable
