# Correction to: Metagenomic analysis of isolation methods of a targeted microbe, *Campylobacter jejuni,* from chicken feces with high microbial contamination

**DOI:** 10.1186/s40168-019-0700-z

**Published:** 2019-06-01

**Authors:** Junhyung Kim, Jae-Ho Guk, Seung-Hyun Mun, Jae-Uk An, Hyokeun Song, Jinshil Kim, Sangryeol Ryu, Byeonghwa Jeon, Seongbeom Cho

**Affiliations:** 1 Research Institute for Veterinary Science and College of Veterinary Medicine, Seoul National University, Seoul, Republic of Korea; 2 Department of Food and Animal Biotechnology, Department of Agricultural Biotechnology, Research Institute for Agriculture and Life Sciences, and Center for Food and Bioconvergence, Seoul National University, Seoul, Republic of Korea; 3 School of Public Health, University of Alberta, Edmonton, Alberta Canada

**Correction to: Microbiome**

**https://doi.org/10.1186/s40168-019-0680-z**

Following publication of the original article [1], the authors reported an error in Fig. 2. The correct figure is shown below.

**Fig. 2:**
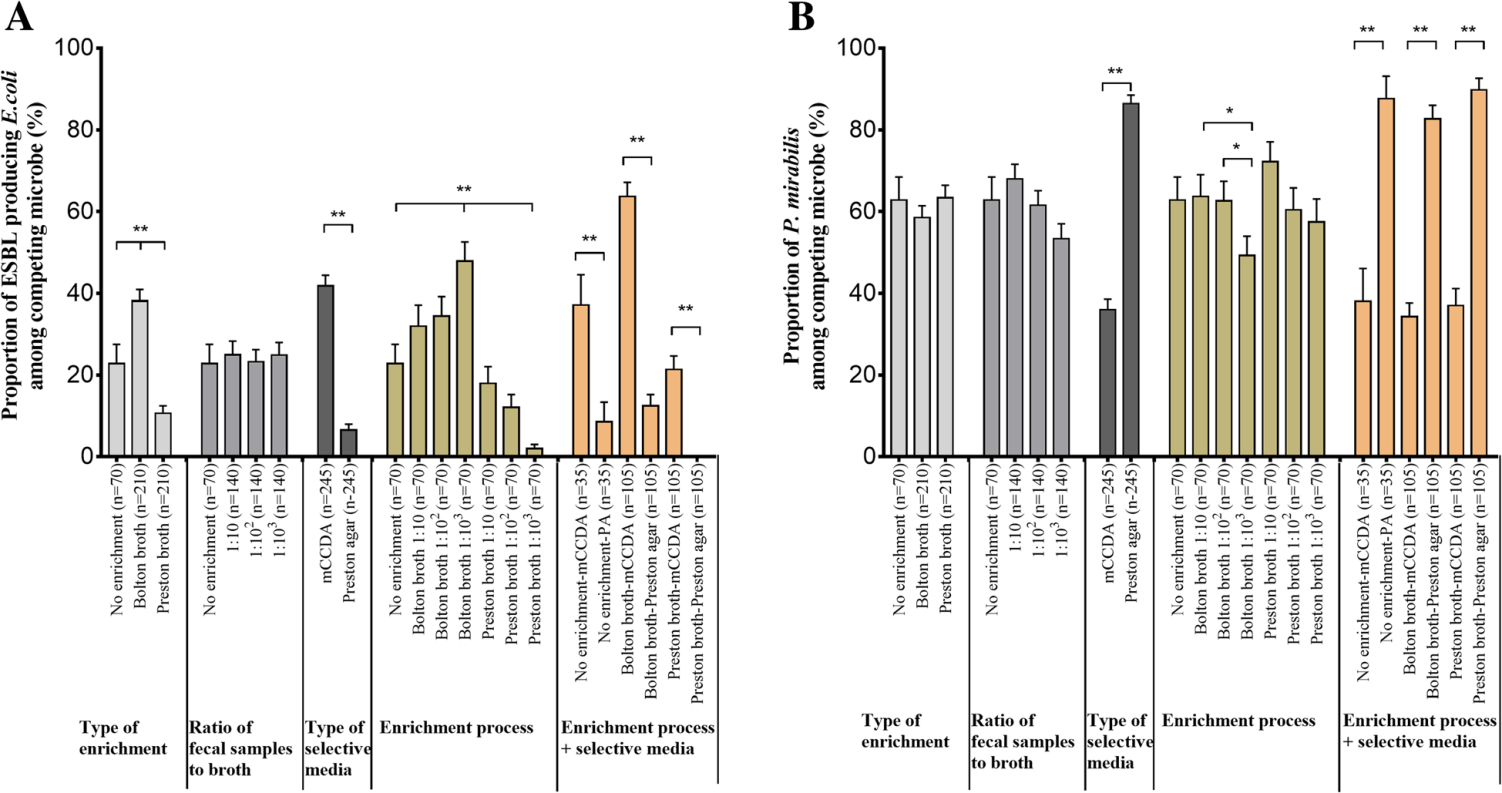
Proportion of competing microbes according to isolation procedures of *C. jejuni* based on culture-dependent tools. Proportion of **a** ESBL-producing *E. coli* and **b** *P. mirabilis* among competing microbes according to the isolation procedure of *C. jejuni* (mean ± SEM). Significance was determined by *t* test and one-way ANOVA. The proportion of ESBL-producing *E. coli* was significantly different according to the isolation procedure including the type of enrichment broth, selective media, and the combination of different enrichment broths, the ratio of sample-to-enrichment broth, and selective media. The proportion of *P. mirabilis* was significantly different according to the isolation procedure including the type of selective media and combination of different enrichment broths, the ratio of sample to enrichment broth, and selective agars. *p < 0.05, **p < 0.01
