# Supplementary material for: Metagenomic analysis of isolation methods of a targeted microbe, Campylobacter jejuni, from chicken feces with high microbial contamination
Source: Microbiome. 2019 Apr 25;7:67. doi: 10.1186/s40168-019-0680-z (PMC6485176; doi:10.1186/s40168-019-0680-z)
Supplement: Supplementary file 1 — Table S1. Information of samples and isolation results of C. jejuni. CJ: positive for C. jejuni in culture-based results, Blank: negative for C. jejuni in culture-based results. Gray color: samples used for microbial community analysis. A total of 54 samples (at least seven samples per process) were used for microbial community analysis. [file 40168_2019_680_MOESM1_ESM.docx]

**Supplementary Table 1. Information of samples and isolation results of *C. jejuni.***

| Enrichment type | | No enrichment | | Bolton broth | | | | | | Preston broth | | | | | | | |
| --- | --- | --- | --- | --- | --- | --- | --- | --- | --- | --- | --- | --- | --- | --- | --- | --- | --- |
| Ratio | | - | | 1:10 | | 1:100 | | 1:1000 | | 1:10 | | 1:100 | | | 1:1000 | | |
| Selective media | | mCCDA | Preston  agar | mCCDA | Preston  agar | mCCDA | Preston  agar | mCCDA | Preston  agar | mCCDA | Preston  agar | | mCCDA | Preston  agar | mCCDA | Preston  agar |  |
| Sample | 1 |  | CJ |  |  |  |  |  |  |  |  | | CJ |  | CJ |  |  |
|  | 2 |  |  |  |  |  |  |  |  |  |  | |  |  | ^a^ CJ |  |  |
|  | 3 |  |  |  |  |  |  |  |  |  |  | |  |  | CJ |  |  |
|  | 4 |  |  |  |  |  |  |  |  |  |  | |  |  | CJ |  |  |
|  | 5 | CJ | CJ |  |  |  |  |  |  |  |  | |  |  | CJ |  |  |
|  | 6 | CJ | CJ |  |  |  |  |  |  |  |  | | CJ |  | CJ |  |  |
|  | 7 |  | CJ |  |  |  |  |  |  |  |  | |  |  | CJ |  |  |
|  | 8 | CJ | CJ |  |  |  |  |  |  |  |  | | CJ |  | CJ |  |  |
|  | 9 | CJ | CJ |  |  |  |  |  |  |  |  | | CJ |  | CJ |  |  |
|  | 10 | CJ | CJ |  |  |  |  |  |  |  |  | |  |  | CJ |  |  |
|  | 11 | CJ | CJ |  |  |  |  |  |  |  |  | |  |  | CJ |  |  |
|  | 12 |  | CJ |  |  |  |  |  |  |  |  | | CJ |  | CJ |  |  |
|  | 13 | CJ | CJ |  |  |  |  |  |  |  |  | | CJ |  | CJ |  |  |
|  | 14 | CJ | CJ |  |  |  |  |  |  |  |  | | CJ |  | CJ |  |  |
|  | 15 | CJ |  |  |  |  |  |  |  |  |  | |  |  |  |  |  |
|  | 16 | CJ |  |  |  |  |  |  |  |  |  | |  |  | CJ |  |  |
|  | 17 | CJ | CJ |  |  |  |  |  |  |  |  | |  |  | CJ |  |  |
|  | 18 |  |  |  |  |  |  |  |  |  |  | |  |  | CJ |  |  |
|  | 19 | CJ | CJ |  |  |  |  |  |  |  |  | | CJ |  | CJ | CJ |  |
|  | 20 |  |  |  |  |  |  |  |  |  |  | | CJ |  | CJ |  |  |
|  | 21 |  | CJ |  |  |  |  |  |  |  |  | |  |  | CJ |  |  |
|  | 22 | CJ |  |  |  |  |  |  |  |  |  | |  |  | CJ |  |  |
|  | 23 | CJ | CJ |  |  |  |  |  |  |  |  | |  |  | CJ | CJ |  |
|  | 24 | CJ | CJ |  |  |  |  |  |  |  |  | | CJ |  | CJ |  |  |
|  | 25 |  | CJ |  |  |  |  |  |  |  |  | |  |  | CJ |  |  |
|  | 26 |  |  |  |  |  |  |  |  |  |  | |  |  | CJ |  |  |
|  | 27 |  |  |  |  |  |  |  |  |  |  | |  |  | CJ |  |  |
|  | 28 |  |  |  |  |  |  |  |  |  |  | | CJ |  | CJ |  |  |
|  | 29 |  |  |  |  |  |  |  |  |  |  | |  |  | CJ |  |  |
|  | 30 | CJ | CJ |  |  |  |  |  |  |  |  | |  |  | CJ |  |  |
|  | 31 |  |  |  |  |  |  |  |  |  |  | |  |  | CJ |  |  |
|  | 32 |  |  |  |  |  |  |  |  |  |  | |  |  | CJ |  |  |
|  | 33 |  |  |  |  |  |  |  |  |  |  | |  |  | CJ |  |  |
|  | 34 | CJ |  |  |  |  |  |  |  |  |  | |  |  | CJ |  |  |
|  | 35 |  | CJ |  |  |  |  |  |  |  |  | |  |  | CJ |  |  |
|  | Total | 17/35  (48.6%) | 19/35  (54.3%) | 0/35  (0%) | 0/35  (0%) | 0/35  (0%) | 0/35  (0%) | 0/35  (0%) | 0/35  (0%) | 0/35  (0%) | 0/35  (0%) | | 11/35  (31.4%) | 0/35  (0%) | 34/35  (97.1%) | 2/35  (5.7%) |  |

CJ: positive for *C. jejuni* in culture-based results, Blank: negative for *C. jejuni* in culture-based results,

Gray color: samples used for microbial community analysis. A total of 54 samples (at least seven samples per process) were used for microbial community analysis.

**Supplementary Table 2. Primer list for polymerase chain reaction (PCR), quantitative PCR, and bacterial DNA amplification in this study.**

|  | **Target microbe** | **Target** | **Size** | **Primer** | **Sequence (5’-3’)** | **Reference** |
| --- | --- | --- | --- | --- | --- | --- |
| PCR | *C. jejuni* | *hipO* | 323 bp | CJ-F | ACTTCTTTATTGCTTGCTGC | [42] |
|  |  |  |  | CJ-R | GCCACAACAAGTAAAGAAGC |  |
|  | *C. coli* | *glyA* | 126 bp | CC-F | GTAAAACCAAAGCTTATCGTG | [42] |
|  |  |  |  | CC-R | TCCAGCAATGTGTGCAATG |  |
|  | *Campylobacter* spp. | 23S rRNA | 650 bp | 23s-F | TATACCGGTAAGGAGTGCTGGAG | [42] |
|  |  |  |  | 23s-R | ATCAATTAACCTTCGAGCACCG |  |
|  | *E. coli* | *MalB* promoter | 585 bp | Eco-F | GACCTCGGTTTAGTTCACAGA | [44] |
|  |  |  |  | Eco-R | CACACGCTGACGCTGACCA |  |
|  | *E. faecium* | - | 658 bp | mFM-F | TTGAGGCAGACCAGATTGACG | [43] |
|  |  |  |  | mFM-R | TATGACAGCGACTCCGATTCC |  |
|  | *E. faecalis* | - | 941 bp | mFL-F | ATCAAGTACAGTTAGTCTTTATTAG | [43] |
|  |  |  |  | mFL-R | ACGATTCAAAGCTAACTGAATCAGT |  |
|  | *Enterococcus* spp*.* | 16S rRNA | 320bp | mENT-F | GGATTAGATACCCTGGTAGTCC | [43] |
|  |  |  |  | mENT-R | TCGTTGCGGGACTTAACCCAAC |  |
| Bacterial DNA amplification | 16S V3-V4 region | V3-V4 region |  | MiSeq 341F | TCGTCGGCAGCGTCAGATGTGTATAAGAGACAGCCTACGGGNGGCWGCAG | [47] |
|  |  |  |  | Miseq 805R | GTCTCGTGGGCTCGGAGATGTGTATAAGAGACAGGACTACHVGGGTATCTAATCC |  |
| Quatitative PCR | *C. jejuni* | *hipO* | 123bp | qCJ-F | AATGCACAAATTTGCCTTATAAAAGC | [55] |
|  |  |  |  | qCJ-R | TNCCATTAAAATTCTGACTTGCTAAATA |  |
|  |  | probe |  | qCJ-probe | FAM-ACATACTACTTCTTTATTGCTTG-BHQ1 |  |
