# Supplementary material for: Metagenomic analysis of isolation methods of a targeted microbe, Campylobacter jejuni, from chicken feces with high microbial contamination
Source: Microbiome. 2019 Apr 25;7:67. doi: 10.1186/s40168-019-0680-z (PMC6485176; doi:10.1186/s40168-019-0680-z)
Supplement: Supplementary file 2 — Table S2. Primer list for polymerase chain reaction (PCR), quantitative PCR, and bacterial DNA amplification in this study. (DOCX 16 kb) [file 40168_2019_680_MOESM2_ESM.docx]

**Additional file 2: Table S2. Primer list for polymerase chain reaction (PCR), quantitative PCR, and bacterial DNA amplification in this study.**

|  | **Target microbe** | **Target** | **Size** | **Primer** | **Sequence (5’-3’)** | **Reference** |
| --- | --- | --- | --- | --- | --- | --- |
| PCR | *C. jejuni* | *hipO* | 323 bp | CJ-F | ACTTCTTTATTGCTTGCTGC | [42] |
|  |  |  |  | CJ-R | GCCACAACAAGTAAAGAAGC |  |
|  | *C. coli* | *glyA* | 126 bp | CC-F | GTAAAACCAAAGCTTATCGTG | [42] |
|  |  |  |  | CC-R | TCCAGCAATGTGTGCAATG |  |
|  | *Campylobacter* spp. | 23S rRNA | 650 bp | 23s-F | TATACCGGTAAGGAGTGCTGGAG | [42] |
|  |  |  |  | 23s-R | ATCAATTAACCTTCGAGCACCG |  |
|  | *E. coli* | *MalB* promoter | 585 bp | Eco-F | GACCTCGGTTTAGTTCACAGA | [44] |
|  |  |  |  | Eco-R | CACACGCTGACGCTGACCA |  |
|  | *E. faecium* | - | 658 bp | mFM-F | TTGAGGCAGACCAGATTGACG | [43] |
|  |  |  |  | mFM-R | TATGACAGCGACTCCGATTCC |  |
|  | *E. faecalis* | - | 941 bp | mFL-F | ATCAAGTACAGTTAGTCTTTATTAG | [43] |
|  |  |  |  | mFL-R | ACGATTCAAAGCTAACTGAATCAGT |  |
|  | *Enterococcus* spp*.* | 16S rRNA | 320bp | mENT-F | GGATTAGATACCCTGGTAGTCC | [43] |
|  |  |  |  | mENT-R | TCGTTGCGGGACTTAACCCAAC |  |
| Bacterial DNA amplification | 16S V3-V4 region | V3-V4 region |  | MiSeq 341F | TCGTCGGCAGCGTCAGATGTGTATAAGAGACAGCCTACGGGNGGCWGCAG | [47] |
|  |  |  |  | Miseq 805R | GTCTCGTGGGCTCGGAGATGTGTATAAGAGACAGGACTACHVGGGTATCTAATCC |  |
| Quatitative PCR | *C. jejuni* | *hipO* | 123bp | qCJ-F | AATGCACAAATTTGCCTTATAAAAGC | [55] |
|  |  |  |  | qCJ-R | TNCCATTAAAATTCTGACTTGCTAAATA |  |
|  |  | probe |  | qCJ-probe | FAM-ACATACTACTTCTTTATTGCTTG-BHQ1 |  |
