# Supplementary figures and images for: Metagenomic analysis of isolation methods of a targeted microbe, Campylobacter jejuni, from chicken feces with high microbial contamination
Source: Microbiome. 2019 Apr 25;7:67. doi: 10.1186/s40168-019-0680-z (PMC6485176; doi:10.1186/s40168-019-0680-z)

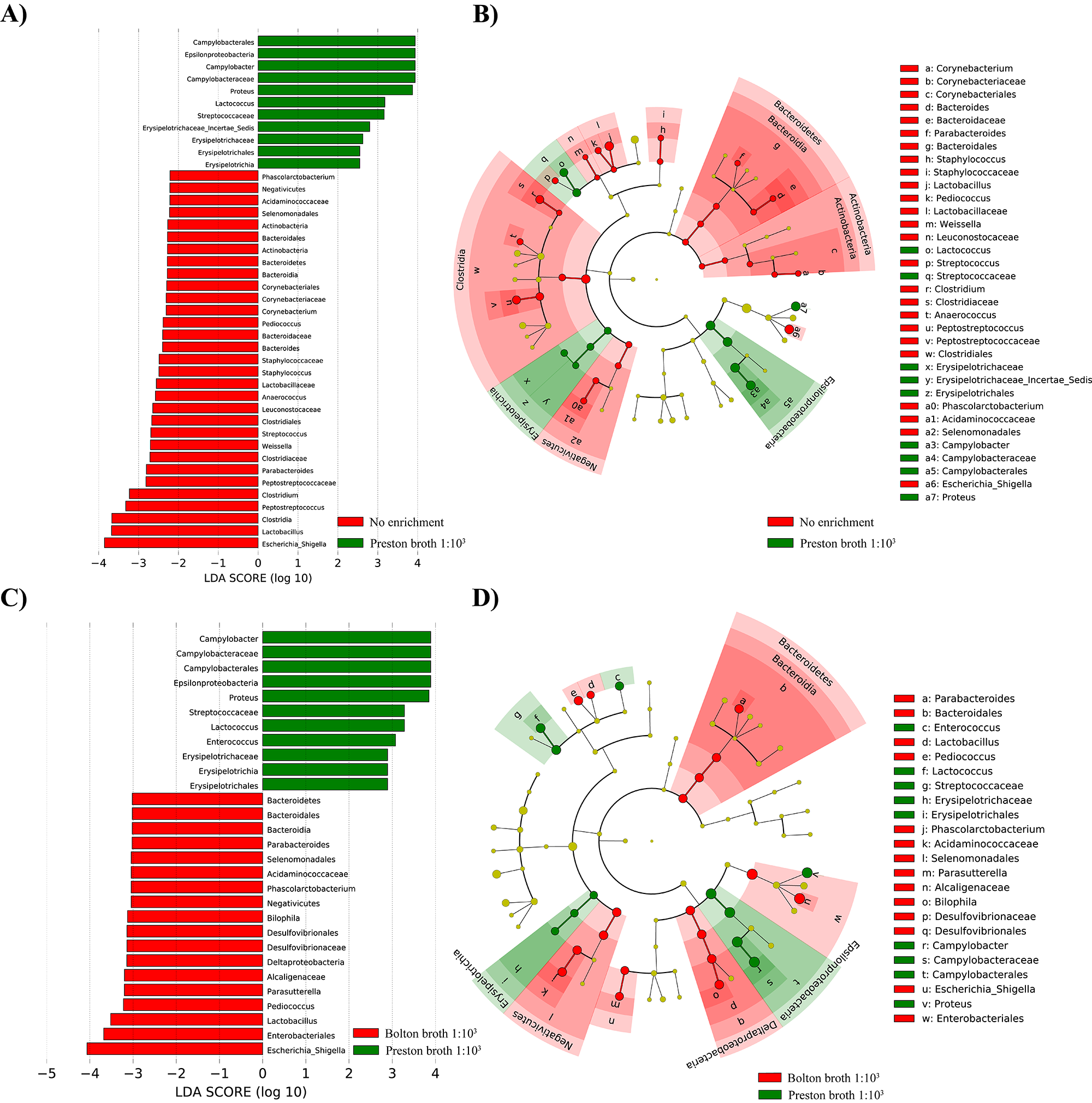

Supplement: Supplementary file 3 — Figure S1. Bacterial taxa that are differentially abundant in the microbial community of fecal samples in each procedure. A) Linear discriminant analysis effect size (LEfSe) and B) taxonomic cladogram between fecal samples enriched in Preston broth at the 1:103 ratio and not undergoing the enrichment process. C) LEfSe and D) taxonomic cladogram between fecal samples enriched in Preston broth and Bolton broth at the 1:103 ratio. The logarithmic linear discriminant analysis score cutoff was set to 2.0. The relative abundance of Campylobacter in fecal samples in 103-Preston broth was significantly higher than in other procedures, while the relative abundance of Escherichia-Shigella was significantly lower than in other procedures. (TIF 935 kb) [file 40168_2019_680_MOESM3_ESM.tif]

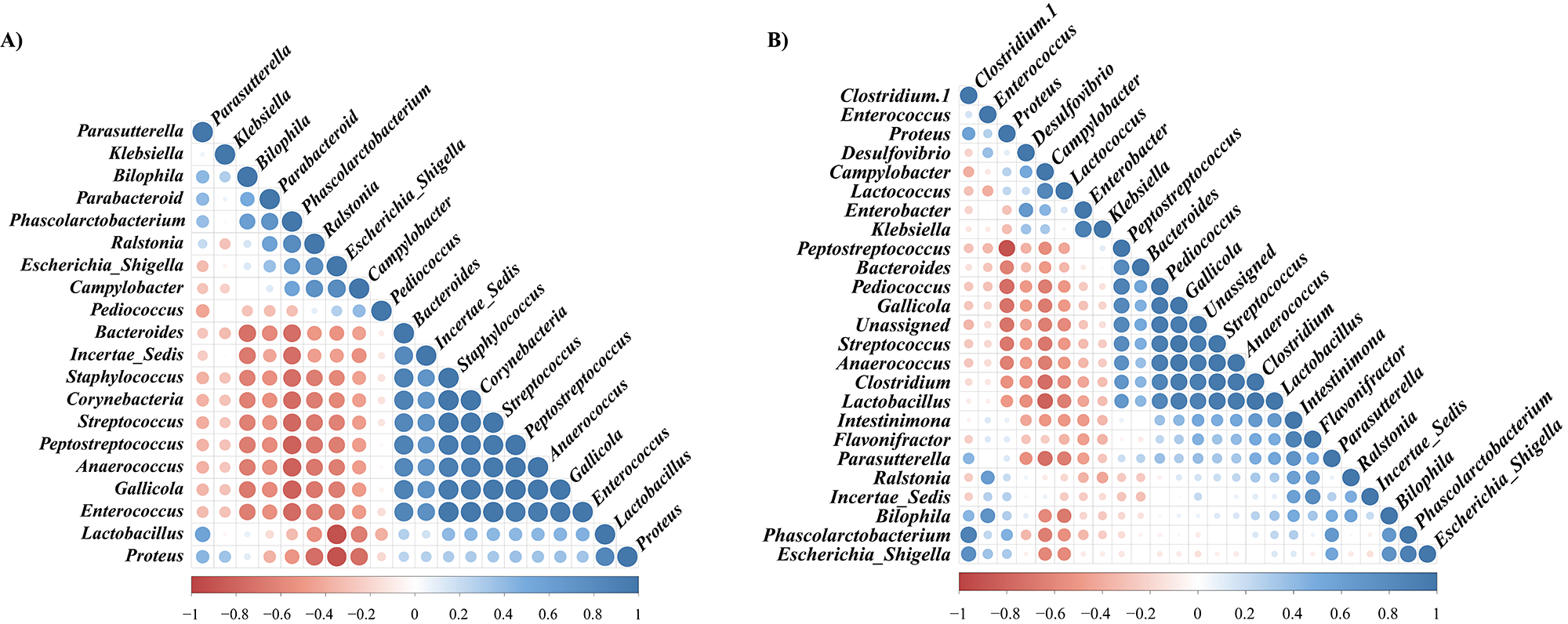

Supplement: Supplementary file 4 — Figure S2. Relationship between microorganisms in microbial community of fecal samples. Correlation plot in A) Bolton broth and B) Preston broth regardless of the ratio of sample- to-enrichment broth. Campylobacter was negatively correlated with Proteus in Bolton broth, while Campylobacter was negatively correlated with Escherichia-Shigella in Preston broth. (TIF 711 kb) [file 40168_2019_680_MOESM4_ESM.tif]

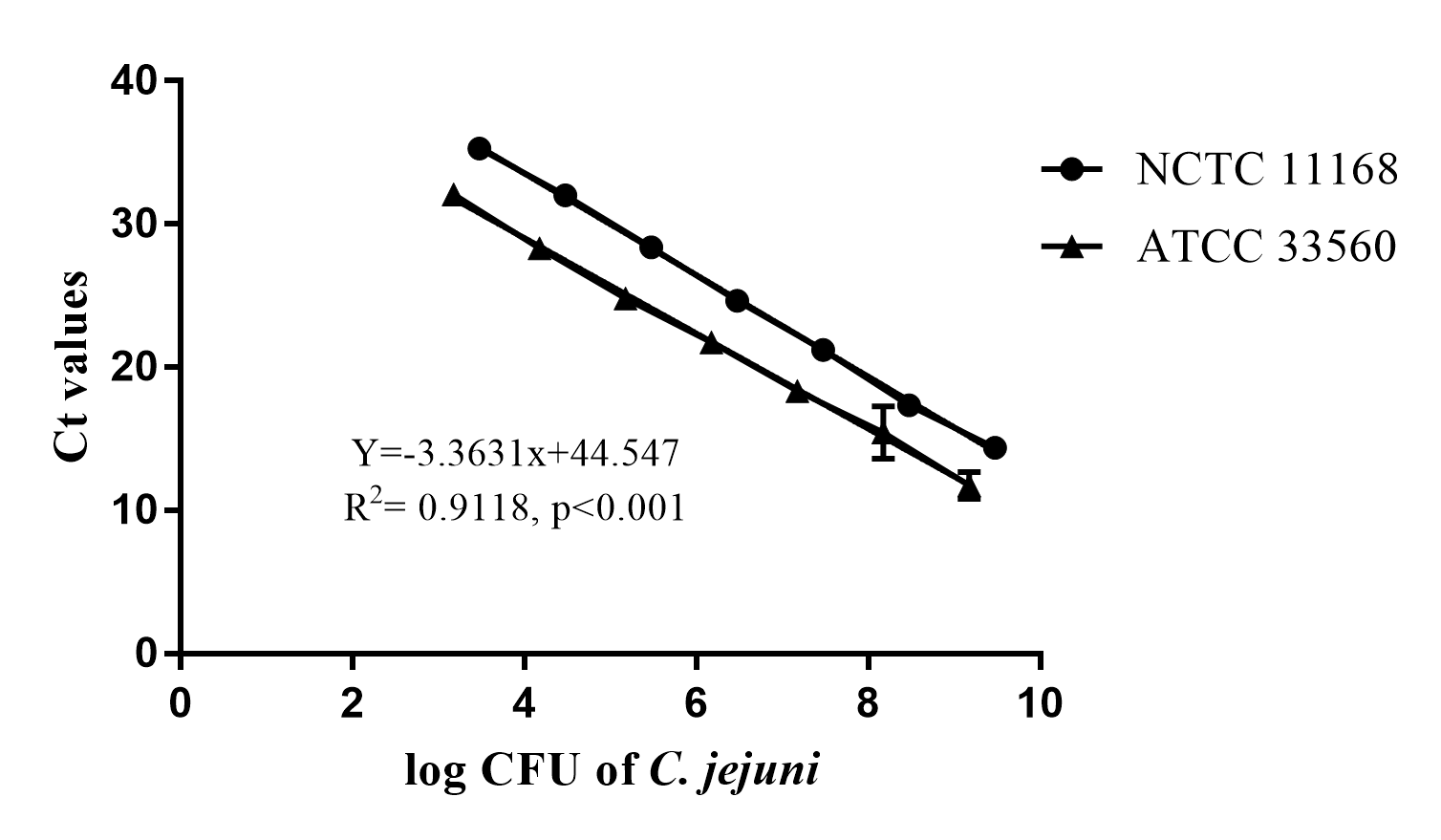

Supplement: Supplementary file 5 — Figure S3. The correlation between colony forming-units and cycle threshold (Ct) values of C. jejuni standard strains (NCTC 11168 and ATCC 33560). (TIF 155 kb) [file 40168_2019_680_MOESM5_ESM.tif]
